# Cohort study of growth patterns by gestational age in preterm infants developing morbidity

**DOI:** 10.1136/bmjopen-2016-012872

**Published:** 2016-11-17

**Authors:** S Klevebro, P Lundgren, U Hammar, L E Smith, M Bottai, M Domellöf, C Löfqvist, B Hallberg, A Hellström

**Affiliations:** 1 Clinical Sciences, Intervention, and Technology, Karolinska Institutet, Stockholm, Sweden; 2 Sachs’ Children and Youth Hospital, South General Hospital, Stockholm, Sweden; 3 Department of Ophthalmology, Institute of Neuroscience and Physiology, Sahlgrenska Academy, University of Gothenburg, Göteborg, Sweden; 4 Unit of Biostatistics, Institute of Environmental Medicine, Karolinska Institutet, Stockholm, Sweden; 5 Department of Ophthalmology, Boston Children's Hospital, Harvard Medical School, Boston, Massachusetts, USA; 6 Department of Clinical Sciences, Paediatrics, Umeå University, Umeå, Sweden; 7 Department of Neonatology, Karolinska University Hospital, Stockholm, Sweden

**Keywords:** NEONATOLOGY, PERINATOLOGY

## Abstract

**Objectives:**

To examine differences in growth patterns in preterm infants developing major morbidities including retinopathy of prematurity (ROP), bronchopulmonary dysplasia (BPD), necrotising enterocolitis (NEC) and intraventricular haemorrhage (IVH).

**Study design:**

Cohort study of 2521 infants born at a gestational age (GA) of 23–30 weeks from 11 level III neonatal intensive care units in USA and Canada, and 3 Swedish population-based cohorts.

**Outcomes:**

Birth weight and postnatal weight gain were examined relative to birth GA and ROP, BPD, NEC and IVH development.

**Results:**

Among infants with a birth GA of 25–30 weeks, birth weight SD score and postnatal weight were lower in those developing ROP and BPD. Infants developing ROP showed lower growth rates during postnatal weeks 7–9 in the 23–24 weeks GA group, during weeks 4–6 in the 25–26 weeks GA group and during weeks 1–5 in the 27–30 weeks GA group. Infants with BPD born at 27–30 weeks GA showed lower growth rates during postnatal weeks 3–5. Infants with NEC had lower growth rates after postnatal week 6 in all GA groups, with no significant differences in birth weight SD score. IVH was not associated with prenatal or postnatal growth.

**Conclusions:**

In this cohort study of extremely preterm infants, we found that the postnatal growth pattern was associated with morbidities such as ROP, BPD and NEC as well as with gestational age at birth.

**Strengths and limitations of this study**

- Large cohort study of detailed growth patterns in extremely and very preterm infants.
- A high number of infants in the earliest gestational ages enabling investigation of differences in growth patterns depending on gestational age at birth.
- Advanced growth models comparing growth patterns generated in collaboration with a team of statisticians.
- This study does not provide an answer to how the presently described associations are mediated.

## Introduction

An important challenge in the care of preterm infants is to reduce the incidence of neonatal and long-term morbidity.^1^ ^2^ It is suggested that growth during the neonatal period should mimic intrauterine growth rates to enable normal organ development, but the optimal growth pattern for preterm infants is not known.^3^ Intrauterine growth rates during the entire neonatal period are rarely achieved in extremely preterm infants.^4^ ^5^

Prenatal factors influence intrauterine maturation and growth, and intrauterine growth restriction (IUGR) increases the risks of abnormal organ development and disease.^6^ Retinopathy of prematurity (ROP) is caused by abnormal postnatal retinal neurovascular development. Previous research has shown that ROP development is related to growth factor levels,^7^ and that low gestational age (GA), associated with very immature retina as a starting point for postnatal growth, is the strongest risk factor for ROP development.^8^ Small for gestational age (SGA) is a risk factor dependent on GA at birth.^9^ Postnatal growth restriction has been used as a marker for ROP risk and is an important component of new surveillance systems developed to refine ROP screening.^10–16^ Bronchopulmonary dysplasia (BPD) results from abnormal postnatal development of pulmonary tissue. It has been suggested that the biological processes that lead to ROP development also play important roles in BPD development.^17^ BPD risk is also reportedly related to IUGR.^6^ ^18^ ^19^ Necrotising enterocolitis (NEC) has been associated with IUGR and SGA, whereas intraventricular haemorrhage (IVH) has not.^6^ ^20^ ^21^

The present study aimed to describe how GA, birth weight and postnatal growth vary with the development of ROP, BPD, NEC and IVH in extremely preterm infants.

## Patients and methods

**Figure 1:**
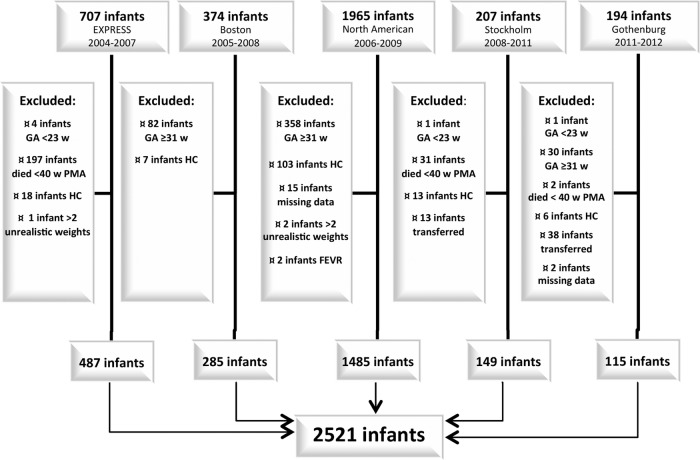
Flow chart of included cohorts. FEVR, familial exudative vitreoretinopathy; GA, gestational age; HC, hydrocephalus; PMA, postmenstrual age; w weeks.

This study used data from five cohorts of preterm infants from Canada, the USA and Sweden (figure 1). The Boston cohort included all infants born before 32 weeks GA and qualified for ROP screening at Brigham and Women's Hospital between 2005 and 2008.^12^ The North American cohort originated from a multicentre study conducted between 2006 and 2009 at 10 level III neonatal intensive care units within the USA and Canada. This study aimed to validate the WINROP screening method.^13^ EXPRESS is a population-based study including all infants born before 27 weeks GA between 2004 and 2007 in Sweden.^14^ The Gothenburg cohort comprised infants born before 32 weeks GA and screened for ROP in Gothenburg between 2011 and 2012.^15^ Our present study also included a previously unpublished population-based cohort of infants born before 27 weeks GA in Stockholm between 2008 and 2011.

Our present study included infants born at a GA of between 23+0 and 30+6 weeks without severe malformations, who survived to a postmenstrual age (PMA) of 40 weeks, and for whom data were available regarding birth weight (BW), maximum ROP stage and/or BPD. Exclusion criteria were hydrocephalus, which gave rise to non-physiological growth patterns, and more than two registered weight measurements that were considered to be unrealistic outliers based on age and relation to adjacent measurements. Data were collected as described in previous studies.^12–15^ Data for infants in the Stockholm cohort were collected from hospital records. GA at birth was based on ultrasound dating or on the date of last menstrual period if no ultrasound had been performed.

Prenatal growth was evaluated as BW compared to the expected weight based on GA and gender. Comparisons were performed using a Swedish foetal growth reference constructed based on intrauterine ultrasound measurements,^22^ which is considered to reflect undisturbed intrauterine growth. For comparison, the descriptive figure for growth also included reference lines from the Olsen growth chart, which is based on American live-born singletons.^23^ Weight measurements were recorded weekly on average, with documentation of the exact date of every measurement. SGA was defined as BW below two SD scores according to the intrauterine growth reference.^22^ ROP screening was performed following routine protocol, with classification according to the international system.^24^ BPD was defined as need for oxygen at a PMA of 36 weeks.^25^ NEC was defined as Bell stage 2b or greater.^26^ IVH registration included any grade of IVH.

### Statistics

All statistical analyses were performed using Stata/IC V.13.1 software (StataCorp LP, College Station, Texas, USA). Data outliers were examined with the use of population and individual residuals in the model and excluded if deemed unrealistic. Level of significance was set at 5%. Descriptive analyses included quantile regression growth models. Differences in BW and birth weight standard deviation score (BWSDS) were estimated using a linear regression model with robust SEs. We applied a mixed effects model including restricted cubic splines (knots at 2, 4, 6 and 10 weeks), with random slope and intercept, to evaluate growth as weight and growth rate over time.^27^ ^28^ Growth rates were calculated using the exponential model suggested by Patel *et al*.^29^ In the mixed effects model, we included growth rates using all weights and calculated the mean between-group differences in weight and growth rate. To describe the overall growth rate in the entire cohort, we calculated the rate from birth weight to weight at 36 weeks PMA. If no measurement at 36 weeks PMA was available, we used the closest value up to 2 weeks prior.

All analyses were initially stratified by gestational week at birth. For the final analyses, participants were divided into three groups by GA: 23–24 weeks, 25–26 weeks and 27–30 weeks. The GA groups were selected based on the results of the 1 week analysis. Postnatal growth results are presented from birth until 36 weeks PMA. All analyses were adjusted for exact gestational age in days, gender and centre. Date of birth was also tested and eliminated as a confounder and therefore not included in the final model. When analysing differences in weight over time and growth rate in cases of ROP and BPD, we included further adjustment for NEC and BWSDS. Analyses were also tested for interaction regarding BWSDS and morbidity and country and morbidity, respectively. No significant association on differences in growth rate were found, and the interaction terms were not included in the final model. Selected adjustments were based on previously known influences, as well as statistical procedures, including backward selection.

### Ethical statement

The Swedish studies were approved by regional ethical review boards (Lund 2004-42; Stockholm 2007-1613312 and 2013-26532; Gothenburg 2013-051-13). The North American studies were approved by institutional review boards at all participating centres (Brigham and Women's Hospital, Boston, Massachusetts, USA; Beaumont Hospital/Associated Retinal Consultants, Oakland University William Beaumont School of Medicine, Royal Oak, Michigan, USA; Beth Israel Deaconess Medical Center, Harvard Medical School, Boston, Massachusetts, USA; Emory University Hospital Midtown, Emory University School of Medicine, Atlanta, Georgia, USA; Morristown Medical Center at Atlantic Health, Atlantic Neonatal Research Institute, Morristown, New Jersey, USA; Mount Sinai Hospital, University of Toronto Faculty of Medicine, Toronto, Ontario, Canada; Nationwide Children’s Hospital, Ohio State University College of Medicine, Columbus; New York-Presbyterian Morgan Stanley Children’s Hospital, Columbia University College of Physicians and Surgeons, New York, New York, USA; North Carolina Children’s Hospital, University of North Carolina School of Medicine, Chapel Hill; UMass Memorial Medical Center, UMass Medical School, Worcester, Massachusetts, USA; Wilford Hall Medical Center, Uniformed Services University of the Health Sciences, Lackland Air Force Base, Texas, USA).

## Results

**Figure 2:**
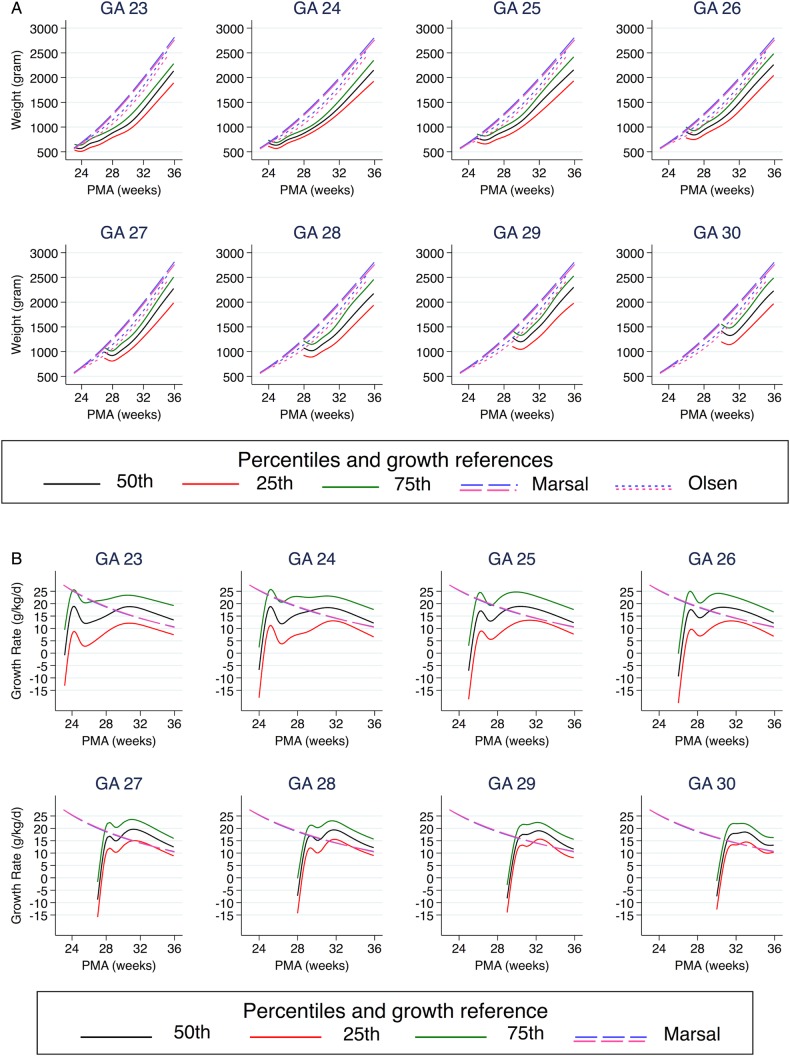
Postnatal weight development and growth rate according to gestational age, illustrated by the 25th, 50th and 75th centiles. (A) Postnatal weight development in grams. (B) Postnatal growth rate in g/kg/day. Growth references 50th centiles from Marsal *et al*^22^ and Olsen *et al.*^23^ Pink denotes female, and blue male.

This study included a total of 2521 infants (figure 1), with a mean of 14.3 weight measurements per infant (median, 11; IQR, 9–16). The last weight measurement was registered at a mean PMA of 37+6 weeks (median, 37+5 weeks; IQR, 36+0–39+5 weeks). Baseline characteristics are demonstrated in table 1. The infants' gestational ages were evenly distributed between the early group (23–26 weeks; 52%) and the later group (27–30 weeks; 48%). The later GA group showed a lower median BWSDS. Infants born at GA 23–26 weeks showed significant postnatal growth restriction, reflected by a reduced mean weight SD score from birth to 36 weeks PMA, with the most pronounced difference among the more immature infants. Figure 2 and table 1 illustrate the growth data. Median weekly growth rates were highest at a PMA of 30 weeks in infants born at GA 23–26 weeks, and at a PMA of 31–34 weeks among infants born at GA 28–30 weeks, corresponding to the fifth week of life.

**Table 1:** Infant characteristics, growth and morbidity by gestational age at birth

	Gestational age at birth (weeks)																	
	23		24		25		26		27		28		29		30		All	
	n	Per cent	n	Per cent	n	Per cent	n	Per cent	n	Per cent	n	Per cent	n	Per cent	n	Per cent	N	Per cent
**Infants**	**117**	**4.6**	**314**	**12.5**	**399**	**15.8**	**482**	**19.1**	**249**	**9.9**	**296**	**11.7**	**323**	**12.8**	**341**	**13.5**	**2521**	**100**
Female sex	58	49.6	137	43.6	190	47.6	229	47.5	119	47.8	139	47.0	134	41.5	162	47.5	1168	46.3
Country																		
NA*	53	45.3	168	53.5	190	47.6	227	47.1	237	95.2	277	93.6	299	92.6	319	93.5	1770	70.2
Sweden	64	54.7	146	46.5	209	52.4	255	52.9	12	4.8	19	6.4	24	7.4	22	6.4	751	29.8
SGA	5	4.3	38	12.1	81	20.3	115	23.9	77	30.9	110	37.2	107	33.1	114	33.4	647	25.7
	Mdn	IQR	Mdn	IQR	Mdn	IQR	Mdn	IQR	Mdn	IQR	Mdn	IQR	Mdn	IQR	Mdn	IQR	Mdn	IQR
BW (g)	595	550–655	675	610–735	770	693–858	890	776–995	989	865–1080	1071	921–1211	1287	1100–1420	1415	1195–1550	915	731–1153
BWSDS (SDS)	−0.4	−1.0–0.4	−0.6	−1.4–0.0	−1.0	−1.7–0.1	−1.0	−2.0–0.2	−1.3	−2.3–0.6	−1.6	−2.6–0.8	−1.3	−2.3–0.4	−1.4	−2.5–0.6	−1.1	−2.0–0.3
Weight at 36 weeks PMA (g)	2072	1847–2263	2123	1932–2319	2126	1881–2377	2190	1905–2415	2244	1922–2501	2179	1958–2500	2320	1973–2582	2206	1902–2506	2157	1909–2391
WSDS at 36 weeks PMA (SDS)	−2.3	−2.9–1.8	−2.2	−2.8–1.6	−2.2	−2.9–1.4	−2.0	−2.9–1.2	−1.6	−2.7–0.8	−1.9	−2.5–0.9	−1.5	−2.6–0.7	−1.8	−2.9–0.9	−1.9	−2.7–1.2
Growth rate† (g/kg/d)	13.7	12.6–15.2	13.8	12.6–15.2	13.8	12.7–15.4	13.8	12.3–15.4	13.7	12.3–15.2	13.3	11.6–14.9	12.4	11.0–14.2	11.8	9.8–13.9	13.3	11.8–15.0
MAX rate (g/kg/d)	18.9	11.5–23.6	18.3	12.2–23.8	19.7	13.1–25.6	20.3	13.4–25.6	21.7	17.8–25.1	20.5	16.9–23.8	20.4	16.6–23.7	19.0	15.7–22.7	17.7	11.3–22.7
PMA @MAX	30		30		30		30		31		32		33		34		31	
	n	Per cent	n	Per cent	n	Per cent	n	Per cent	n	Per cent	n	Per cent	n	Per cent	n	Per cent	N	Per cent
ROP‡																		
No	9	7.7	17	5.4	69	17.3	166	34.4	99	39.8	185	62.5	236	73.1	304	89.1	1085	43.0
ROP 1–2	40	34.2	131	41.9	215	54.0	236	49.0	127	51.0	98	33.1	84	26.0	34	10.0	965	38.3
ROP≥3	68	58.1	165	52.7	114	28.6	80	16.6	23	9.2	13	4.4	3	0.9	3	0.9	471	18.7
Trt	51	43.6	109	34.9	65	16.3	39	8.1	16	6.4	5	1.7	3	0.9	0	0.0	288	11.4
BPD‡	104	88.9	247	79.2	289	72.8	308	64.6	124	49.8	108	36.5	58	18.0	30	8.8	1268	50.5
NEC	9	7.7	47	15.0	33	8.3	42	8.7	25	10.0	32	10.8	27	8.4	15	4.4	230	9.1
All IVH	60	51.3	144	45.9	141	35.3	118	24.5	65	26.1	56	18.9	64	19.8	44	12.9	692	27.4

*North America (USA and Canada).

†Overall growth rate from birth to 36 weeks PMA calculated for 2344 infants.

‡Information regarding ROP was missing for one infant born at a gestational age of 24 weeks, and one infant at 25 weeks. Information regarding BPD was missing from two infants born at a gestational age of 24 weeks, two infants at 25 weeks and five infants born at 26 weeks.

BWSDS, birth weight SD score; BPD, bronchopulmonary dysplasia; IVH, intraventricular haemorrhage; Mdn, median; NEC, necrotising enterocolitis; PMA, postmenstrual age; ROP, retinopathy of prematurity; Trt, treatment for ROP; w, weeks. IQR (25th to 75th centile).

### Morbidity

Table 1 displays the numbers and frequencies of ROP, BPD, NEC and IVH by gestational week. Differences in BWSDS, postnatal weight and growth rate are shown in figured 3 and 4, as well as in the online supplementary appendix.

10.1136/bmjopen-2016-012872.supp1 supplementary appendix

**Figure 3:**
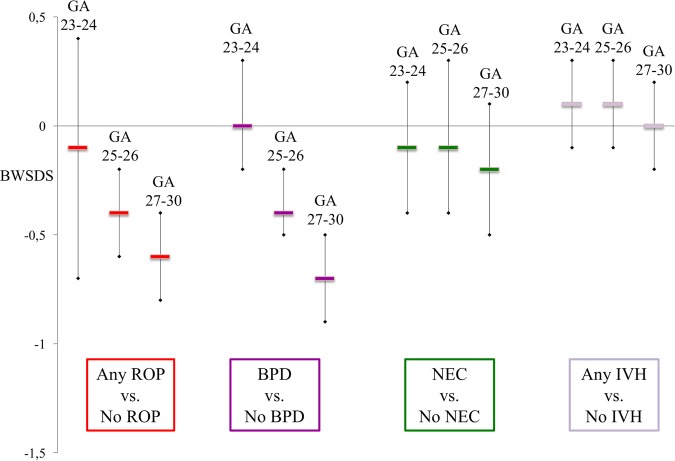
Difference in BWSDS according to gestational age group, between infants with and without respective morbidities. Data are shown as mean and 95% CI for each diagnose. All analyses are adjusted for exact gestational age at birth, gender and centre. BPD, bronchopulmonary dysplasia; BWSDS, birth weight SD score; NEC, necrotising enterocolitis; IVH, intraventricular haemorrhage; ROP, retinopathy of prematurity.

**Figure 4:**
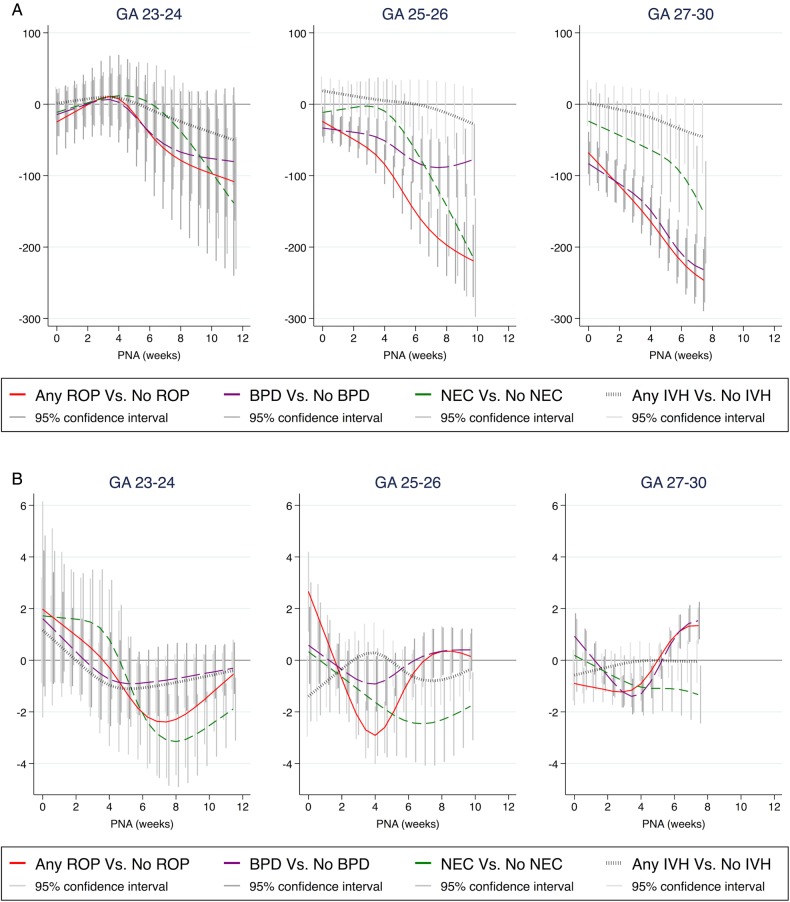
Difference in weight and in growth rate over time according to gestational age group, between infants with and without respective morbidities. (A) The difference in weight in grams. (B) The difference in growth rate in g/kg/day. Time is represented by PNA in weeks. Data are shown as mean and 95% CI for each diagnosis. All analyses are adjusted for exact gestational age at birth, gender and centre. Analyses of differences regarding ROP and BPD are also adjusted for NEC and in growth rate analyses for BWSDS. BPD, bronchopulmonary dysplasia; BWSDS, birth weight SD score; NEC, necrotising enterocolitis; IVH, intraventricular haemorrhage; PNA, postnatal age; ROP, retinopathy of prematurity.

### ROP and growth

Infants developing ROP of any grade showed lower growth rates compared to infants not developing ROP. This difference was noted in the 7–9th weeks of life among infants born at GA 23–24 weeks, and in the 4–6th weeks of life in those born at GA 25–26 weeks. Infants born at GA 27–30 weeks had lower growth rates during their first 5 weeks of life, shifting to higher growth rates starting at the 7th week of life. Among infants born at GA 25–30 weeks, BWSDS was lower in infants developing ROP. Differences at birth were greater among infants born at later GA (see online supplementary appendix figure S1 and table S1).

### BPD and growth

Among those born at GA 27–30 weeks, infants with BPD had lower growth rates in the 4–5th week of life compared to infants without BPD, followed by higher growth rates from the 7th week of life. Among those born at GA 23–24 weeks, infants developing BPD showed significantly lower weight during the 8–12th week of life compared to those without BPD, while these groups did not significantly differ in growth rate. Among those born at GA 25–30 weeks, BWSDS was lower in infants developing BPD. These differences were greater among infants born at later GA, and weight continued to be lower from birth to PMA 36 weeks (see online supplementary appendix figure S2 and table S2).

### NEC and growth

Among infants born at all GAs, those with NEC showed lower growth rates from the 6–7th week of life and during the rest of the studied neonatal period compared to infants without NEC. Infants born at all GAs with and without NEC did not significantly differ in BWSDS (see online supplementary appendix figure S3 and table S3).

### IVH and growth

Analysis of infants with any IVH compared to with no IVH revealed no associations with prenatal or postnatal growth (see online supplementary appendix figure S4).

### SGA and postnatal growth

Compared to infants born at a weight appropriate for their gestational age, those born SGA continued to show lower weights until 36 weeks PMA. For the first 3 weeks of life, SGA infants showed higher growth rates than appropriate for gestational age infants (see online supplementary appendix figure S5).

## Discussion

This study demonstrates differences in postnatal growth patterns in infants developing morbidities and highlights specific postmenstrual weeks with greater differences in growth rates, potentially implicating important windows of vulnerability partly depending on GA. Our present results showed similar patterns of prenatal and postnatal growth restriction between infants developing ROP and BPD. Infants born at a GA of 25–30 weeks who developed ROP and BPD were smaller at birth. Infants born at all GAs who developed ROP had reduced growth rates. Among those born at GA 23–26 weeks, this reduction was most pronounced around the postnatal weeks corresponding to the 31st postmenstrual week. Those born at GA 27–30 weeks and developing ROP and BPD showed an initially reduced growth rate, which shifted to higher growth rates in later weeks. Infants developing NEC and IVH showed a different pattern of prenatal and postnatal growth restriction, indicating a different pathophysiology. The frequency of especially ROP and BPD is high among the most immature infants; 76% of the infants in GA 23–24 weeks developed ROP and BPD, which is important to bear in mind comparing the growth patterns between morbidities (see online supplementary appendix table S4).

To the best of our knowledge, this is the largest detailed study of postnatal weight development among low-gestational age infants, including a large proportion of extremely preterm infants. Stratification by GA rather than birth weight enables consideration of the increased morbidity risks associated with a greater degree of immaturity. The wide range of methods used to evaluate postnatal growth in previous studies makes it difficult to compare results among studies. In 1999, Ehrenkranz *et al*^4^ described the postnatal growth pattern of infants developing morbidity, reporting a mean growth rate (from regaining BW to discharge, death or reaching 2 kg) of 14–16 g/kg/day, increasing with increasing GA. Horbar *et al*^30^ reported growth rates calculated using the two-point exponential model from birth to discharge. The mean growth rates from 2013 in GA 24–26 weeks were 13.0 g/kg/day (95% CI 13.0 to 13.1). Martin *et al*^31^ reported a relationship between lower BWSDS and higher growth rates during the first weeks of life that was similar to the association observed in our cohort. However, Martin *et al*^31^ did not use an exponential growth model to calculate growth rates, resulting in higher rates compared to those found in our study. Our presently used exponential model has been evaluated and deemed most appropriate for calculating growth rates of preterm infants.^29^ Fortes Filho *et al*^32^ described a relationship between severe ROP and proportion of birth weight gained at 6 weeks of age. Their results are consistent with our findings in infants born at a GA of 25–30 weeks. Nyp *et al*^33^ evaluated growth rate (in g/kg from BW to 36 weeks PMA) among 140 infants born before 28 weeks of GA and found no association with BPD. In our substantially larger cohort, we detected associations between BPD and postnatal growth that related to specific postnatal weeks.

Limitations of our present study include a lack of information regarding some perinatal background data preventing any conclusions regarding causality. Notably, our material included no information regarding sepsis. Ehrenkranz *et al*^4^ demonstrated that infants with sepsis have growth pattern similar to those of infants with BPD. Moreover, the use of BWSDS to classify foetal growth restriction could lead to misclassification of infants who are genetically SGA without foetal growth restriction. Additionally, this study included only infants who survived to 40 weeks PMA, which could affect the description of postnatal growth within the entire cohort but not our main outcomes. The combined data set included no information regarding time of NEC. The fact that surgically managed NEC has been previously associated with substantial growth delay,^34^ and the growth patterns demonstrated in our present results suggest that the NEC disease process precedes growth restriction. IVH—which usually occurs early postnatally—does not appear to be strongly related to postnatal growth. Caution is advised interpreting significant results in a study including multiple comparisons. This study examines growth in four morbidities in three strata of GA. The time is handled as a continuum; p values regarding differences in growth rates are presented week by week in the online supplementary appendix.

From this study, it is not possible to determine how the presently described associations are mediated. Analysis of the relationship between nutrition and ROP in the EXPRESS cohort revealed that low energy intake during the first 4 weeks of life was related to increased frequency of severe ROP, after adjustment for several morbidities.^35^ Nutrition practices have most likely been continuously altered during the studied period of time. Including date of birth in the model did not alter the results, but the need to adjust for centre in the final model demonstrates that centre is associated with weight and disease development even adjusted for GA at birth and BWSDS and could be a reflection of differences in perinatal care. Both the pattern of initially reduced and later increased growth rates could be of importance for processes involved in the development of ROP and BPD. The specific weeks showing significantly reduced growth rates corresponded to a PMA of around 31 weeks in infants born at a GA of 23–26 weeks, implicating an especially important period of growth in extremely preterm infants. This is the period of maximum growth rate and represents the transition from growth failure to catch-up growth among infants born at these GAs. Hansen-Pupp *et al*^36^ described increased insulin-like growth factor I (IGF-I) concentrations at PMA 30 weeks, coinciding with the initiation of catch-up growth. Moreover, ROP severity is correlated to IGF-I serum concentrations and duration of low IGF-I concentrations.^7^ ^37^ The reduced growth rates at a PMA of around 30 weeks could be biologically related to initiation of phase II of ROP development, which has been demonstrated to correlate to an increase of the initially low levels of IGF-I.^7^ Lower IGF-I levels in the first postnatal weeks are also associated with BPD among very preterm infants.^17^ It is reasonable to postulate that factors that control somatic growth also influence retina and lung development. ROP is a vascular and neurological disease. Poor neurodevelopment has been related to poor postnatal growth,^38^ ^39^ and postnatal IGF-I concentrations have been associated with brain volume at term.^40^ Neonatal care should aim at optimal growth during the entire neonatal period. Future clinical trials should further investigate the specific periods of slower growth rates and catch-up growth and continue to analyse interactions between growth factors, nutrition, weight gain and morbidities, including neurodevelopmental outcomes. Our present results highlight postnatal growth as a marker for disease, emphasising the importance of monitoring growth and nutrition in the neonatal intensive care unit.

## References

[1] Serenius F, Källén K, Blennow M EXPRESS Group. Neurodevelopmental outcome in extremely preterm infants at 2.5 years after active perinatal care in Sweden. JAMA 2013;309:1810–20. 10.1001/jama.2013.3786 23632725

[2] Stoll BJ, Hansen NI, Bell EF Neonatal outcomes of extremely preterm infants from the NICHD Neonatal Research Network. Pediatrics 2010;126:443–56. 10.1542/peds.2009-2959 20732945 PMC2982806

[3] Agostoni C, Buonocore G, Carnielli VP Enteral nutrient supply for preterm infants: commentary from the European Society of Paediatric Gastroenterology, Hepatology and Nutrition Committee on Nutrition. J Pediatr Gastroenterol Nutr 2010;50:85–91. 10.1097/MPG.0b013e3181adaee0 19881390

[4] Ehrenkranz RA, Younes N, Lemons JA Longitudinal growth of hospitalized very low birth weight infants. Pediatrics 1999;104:280–9. 10.1542/peds.104.2.280 10429008

[5] Stoltz Sjöström E, Öhlund I, Ahlsson F Nutrient intakes independently affect growth in extremely preterm infants: results from a population-based study. Acta Paediatr 2013;102:1067–74. 10.1111/apa.12359 23855971

[6] Zeitlin J, El Ayoubi M, Jarreau PH MOSAIC Research Group. Impact of fetal growth restriction on mortality and morbidity in a very preterm birth cohort. J Pediatr 2010;157:733–9.e1. 10.1016/j.jpeds.2010.05.002 20955846

[7] Hellstrom A, Perruzzi C, Ju M Low IGF-I suppresses VEGF-survival signaling in retinal endothelial cells: direct correlation with clinical retinopathy of prematurity. Proc Natl Acad Sci USA 2001;98:5804–8. 10.1073/pnas.101113998 11331770 PMC33294

[8] Darlow BA, Hutchinson JL, Henderson-Smart DJ Prenatal risk factors for severe retinopathy of prematurity among very preterm infants of The Australian and New Zealand Neonatal Network. Pediatrics 2005;115:990–6. 10.1542/peds.2004-1309 15805375

[9] Lundgren P, Kistner A, Andersson EM Low birth weight is a risk factor for severe retinopathy of prematurity depending on gestational age. PLoS One 2014;9:e109460 10.1371/journal.pone.0109460 25330287 PMC4198133

[10] Löfqvist C, Andersson E, Sigurdsson J Longitudinal postnatal weight and insulin-like growth factor I measurements in the prediction of retinopathy of prematurity. Arch Ophthalmol 2006;124:1711–18. 10.1001/archopht.124.12.1711 17159030

[11] Binenbaum G, Ying GS, Quinn GE A clinical prediction model to stratify retinopathy of prematurity risk using postnatal weight gain. Pediatrics 2011;127:e607–14. 10.1542/peds.2010-2240 21321036 PMC3065141

[12] Wu C, Vanderveen DK, Hellstrom A Longitudinal postnatal weight measurements for the prediction of retinopathy of prematurity. Arch Ophthalmol 2010;128:443–7. 10.1001/archophthalmol.2010.31 20385939 PMC4393744

[13] Wu C, Lofqvist C, Smith LE Importance of early postnatal weight gain for normal retinal angiogenesis in very preterm infants: a multicenter study analyzing weight velocity deviations for the prediction of retinopathy of prematurity. Arch Ophthalmol 2012;130:992–9. 22491391 10.1001/archophthalmol.2012.243 PMC4059056

[14] Lundgren P, Stoltz Sjöström E, Domellöf M WINROP identifies severe retinopathy of prematurity at an early stage in a nation-based cohort of extremely preterm infants. PLoS One 2013;8:e73256 10.1371/journal.pone.0073256 24069180 PMC3771982

[15] Lundgren P, Wilde Å, Löfqvist C Weight at first detection of retinopathy of prematurity predicts disease severity. Br J Ophthalmol 2014;98:1565–9. 10.1136/bjophthalmol-2014-304905 24963022 PMC4389626

[16] Eckert GU, Fortes Filho JB, Maia M A predictive score for retinopathy of prematurity in very low birth weight preterm infants. Eye (Lond) 2012;26:400–6. 10.1038/eye.2011.334 22193874 PMC3298990

[17] Löfqvist C, Hellgren G, Niklasson A Low postnatal serum IGF-I levels are associated with bronchopulmonary dysplasia (BPD). Acta Paediatr 2012;101:1211–16. 10.1111/j.1651-2227.2012.02826.x 22924869 PMC3569611

[18] Bose C, Van Marter LJ, Laughon M Fetal growth restriction and chronic lung disease among infants born before the 28th week of gestation. Pediatrics. 2009;124:e450–8. 10.1542/peds.2008-3249 19706590 PMC2891899

[19] Eriksson L, Haglund B, Odlind V Perinatal conditions related to growth restriction and inflammation are associated with an increased risk of bronchopulmonary dysplasia. Acta Paediatr 2015;104:259–63. 10.1111/apa.12888 25469645

[20] Garite TJ, Clark R, Thorp JA Intrauterine growth restriction increases morbidity and mortality among premature neonates. Am J Obstet Gynecol 2004;191:481–7. 10.1016/j.ajog.2004.01.036 15343225

[21] March MI, Gupta M, Modest AM Maternal risk factors for neonatal necrotizing enterocolitis. J Matern Fetal Neonatal Med 2015;28:1285–90. 10.3109/14767058.2014.951624 PMC4457698 25162307

[22] Marsál K, Persson PH, Larsen T Intrauterine growth curves based on ultrasonically estimated foetal weights. Acta Paediatr 1996;85:843–8. 10.1111/j.1651-2227.1996.tb14164.x 8819552

[23] Olsen IE, Groveman SA, Lawson ML New intrauterine growth curves based on United States data. Pediatrics 2010;125:e214–24. 10.1542/peds.2009-0913 20100760

[24] International Committee for the Classification of Retinopathy of Prematurity. The International Classification of Retinopathy of Prematurity revisited. Arch Ophthalmol 2005;123:991–9. 10.1001/archopht.123.7.991 16009843

[25] Jobe AH, Bancalari E Bronchopulmonary dysplasia. Am J Respir Crit Care Med. 2001;163:1723–9. 10.1164/ajrccm.163.7.2011060 11401896

[26] Walsh MC, Kliegman RM Necrotizing enterocolitis: treatment based on staging criteria. Pediatr Clin North Am 1986;33:179–201. 10.1016/S0031-3955(16)34975-6 3081865 PMC7131118

[27] Durrleman S, Simon R Flexible regression models with cubic splines. Stat Med 1989;8:551–61. 10.1002/sim.4780080504 2657958

[28] Laird NM, Ware JH Random-effects models for longitudinal data. Biometrics 1982;38:963–74. 10.2307/2529876 7168798

[29] Patel AL, Engstrom JL, Meier PP Accuracy of methods for calculating postnatal growth velocity for extremely low birth weight infants. Pediatrics 2005;116:1466–73. 10.1542/peds.2004-1699 16322172

[30] Horbar JD, Ehrenkranz RA, Badger GJ Weight growth velocity and postnatal growth failure in infants 501 to 1500 grams: 2000–2013. Pediatrics 2015;136:e84–92. 10.1542/peds.2015-0129 26101360

[31] Martin CR, Brown YF, Ehrenkranz RA Nutritional practices and growth velocity in the first month of life in extremely premature infants. Pediatrics 2009;124:649–57. 10.1542/peds.2008-3258 19651583 PMC2859427

[32] Fortes Filho JB, Bonomo PP, Maia M Weight gain measured at 6 weeks after birth as a predictor for severe retinopathy of prematurity: study with 317 very low birth weight preterm babies. Graefes Arch Clin Exp Ophthalmol 2009;247:831–6. 10.1007/s00417-008-1012-3 19052770

[33] Nyp MF, Taylor JB, Norberg M Impaired growth at birth and bronchopulmonary dysplasia classification: beyond small for gestational age. Am J Perinatol 2015;32:75–82. 10.1055/s-0034-1376181 24839148

[34] Hintz SR, Kendrick DE, Stoll BJ Neurodevelopmental and growth outcomes of extremely low birth weight infants after necrotizing enterocolitis. Pediatrics 2005;115:696–703. 10.1542/peds.2004-0569 15741374

[35] Stoltz Sjöström E, Lundgren P, Öhlund I Low energy intake during the first 4 weeks of life increases the risk for severe retinopathy of prematurity in extremely preterm infants. Arch Dis Child Fetal Neonatal Ed 2016;101:F108–13. 10.1136/archdischild-2014-306816 25678632 PMC4789715

[36] Hansen-Pupp I, Löfqvist C, Polberger S Influence of insulin-like growth factor I and nutrition during phases of postnatal growth in very preterm infants. Pediatr Res 2011;69(Pt 1):448–53. 10.1203/PDR.0b013e3182115000 21263374

[37] Hellström A, Engström E, Hård AL Postnatal serum insulin-like growth factor I deficiency is associated with retinopathy of prematurity and other complications of premature birth. Pediatrics 2003;112:1016–20. 10.1542/peds.112.5.1016 14595040

[38] Ehrenkranz RA, Dusick AM, Vohr BR Growth in the neonatal intensive care unit influences neurodevelopmental and growth outcomes of extremely low birth weight infants. Pediatrics 2006;117:1253–61. 10.1542/peds.2005-1368 16585322

[39] Franz AR, Pohlandt F, Bode H Intrauterine, early neonatal, and postdischarge growth and neurodevelopmental outcome at 5.4 years in extremely preterm infants after intensive neonatal nutritional support. Pediatrics 2009;123:e101–9. 10.1542/peds.2008-1352 19117831

[40] Hansen-Pupp I, Hövel H, Hellström A Postnatal decrease in circulating insulin-like growth factor-I and low brain volumes in very preterm infants. J Clin Endocrinol Metab 2011;96:1129–35. 10.1210/jc.2010-2440 21289247

