# Supplementary material for: Cohort study of growth patterns by gestational age in preterm infants developing morbidity
Source: BMJ Open. 2016 Nov 17;6(11):e012872. doi: 10.1136/bmjopen-2016-012872 (PMC5128893; doi:10.1136/bmjopen-2016-012872)
Supplement: supplementary appendix [file bmjopen-2016-012872supp_appendix.pdf]

Table of Contents

2-3. Figure S1A, S1B and Table S1 (Growth of Infants With Any ROP vs. No ROP)

4-5. Figure S2A, S2B and Table S2 (Growth of Infants With BPD vs. No BPD)

6-7. Figure S3A, S3B and Table S3 (Growth of Infants With NEC vs. No NEC)

8. Figure S4 (Growth of Infants With Any IVH vs. No IVH)

9. Figure S5A and S5B (Growth of Small for Gestational Age Infants)

10. Table S4 (Multiple Morbidities by Gestational Age)

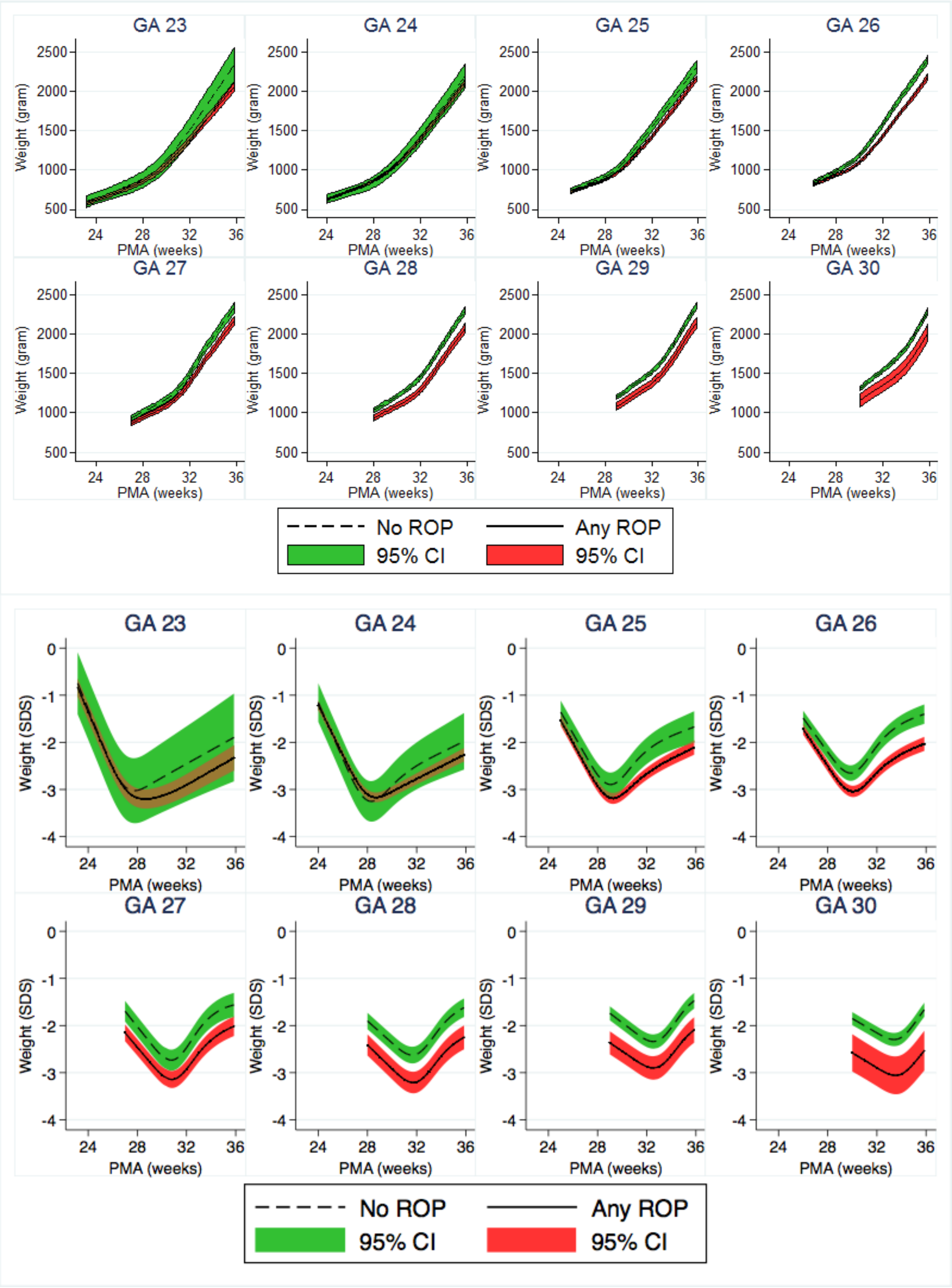

Figure S1. Postnatal Weight Development According to Gestational Age at Birth in Infants With and Without Retinopathy of Prematurity (ROP).

Panel A shows postnatal weight development in grams. Panel B shows development in weight standard deviation score (SDS) according to Marsal[22]. Data are shown as mean and 95% confidence interval.

Gestational Age at birth (weeks)						
week of life	23-24		25-26		27-30	
	diff (g/kg/d)	p	diff (g/kg/d)	p	diff (g/kg/d)	p
**1**	2.0	*0.36*	2.7	*<0.001*	-0.9	*0.04*
**2**	1.5	*0.33*	1.0	*0.08*	-1.0	*<0.001*
**3**	1.0	*0.43*	-0.7	*0.10*	-1.2	*<0.001*
**4**	0.4	*0.77*	-2.3	*<0.001*	-1.2	*<0.001*
**5**	-0.3	*0.85*	-2.9	*<0.001*	-0.9	*0.007*
**6**	-1.2	*0.31*	-2.1	*<0.001*	0.0	*0.99*
**7**	-2.1	*0.04*	-0.8	*0.07*	0.9	*0.003*
**8**	-2.4	*0.03*	0.0	*0.96*	1.3	*<0.001*
**9**	-2.3	*0.04*	0.3	*0.50*		
**10**	-1.9	*0.06*	0.3	*0.51*		
**11**	-1.4	*0.11*				
**12**	-0.8	*0.27*				

Table S1. Difference in Growth Rate Over Time According to Gestational Age Group, Between Infants With and Without Retinopathy of Prematurity (ROP).

Growth rate is reported in grams/kilogram/day. Data are shown as mean and 95% confidence interval. Analyses are adjusted for exact gestational age at birth, gender, center, necrotizing enterocolitis (NEC), and birth weight standard deviation score (BWSDS).

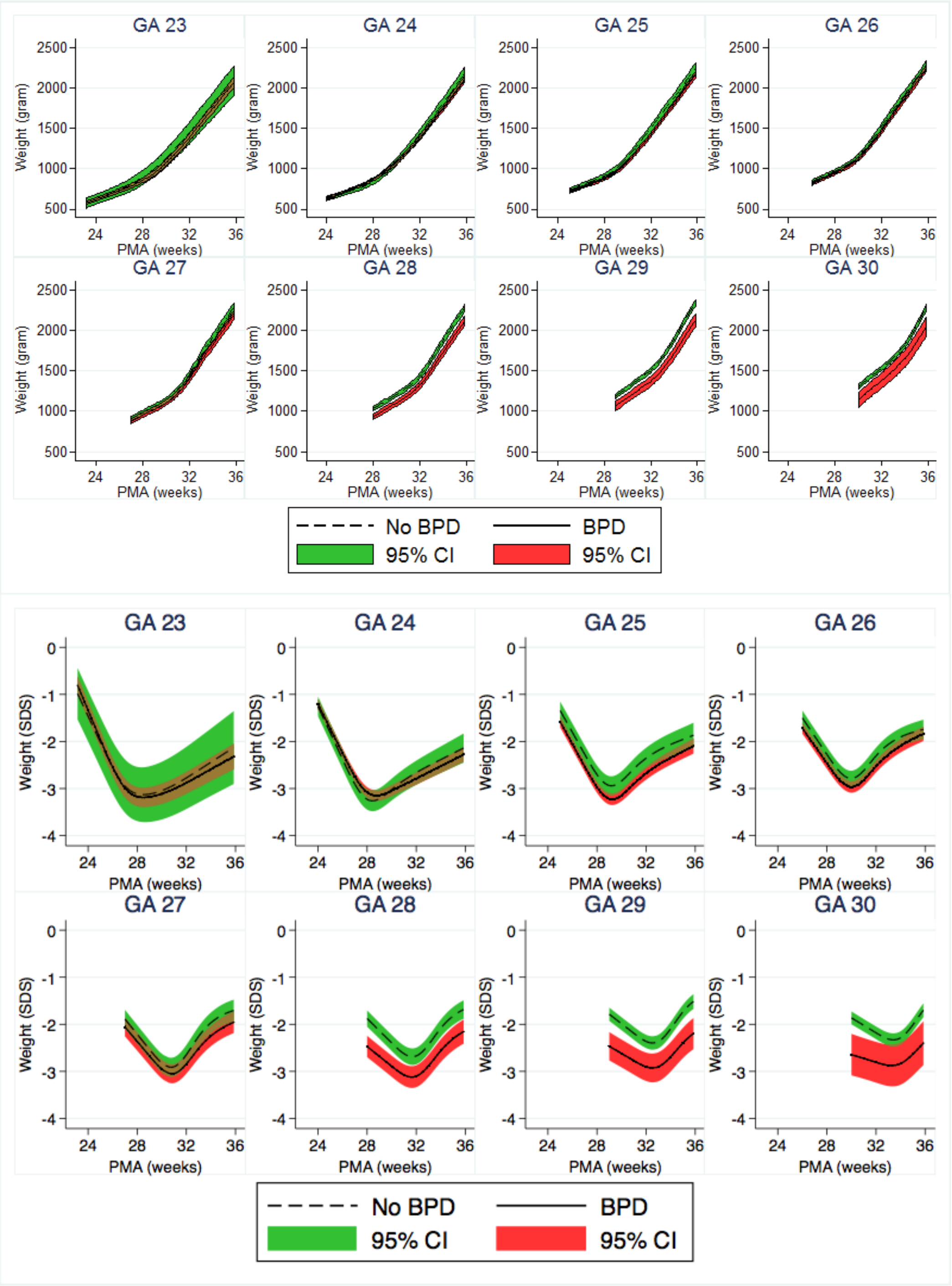

Figure S2. Postnatal Weight Development According to Gestational Age at Birth in Infants With and Without Bronchopulmonary Dysplasia.

Bronchopulmonary dysplasia (BPD) is defined as requiring O_2 at 36 weeks postmenstrual age (PMA). Panel A shows postnatal weight development in grams. Panel B shows development in weight standard deviation score (SDS) according to Marsal[22]. Data are shown as mean and 95% confidence interval.

Gestational Age at birth (weeks)						
**week of life**	**23-24**		**25-26**		**27-30**	
	diff (g/kg/d)	p	diff (g/kg/d)	p	diff (g/kg/d)	p
**1**	1.6	*0.23*	2.7	*0.45*	0.9	*0.04*
**2**	1.0	*0.31*	1.0	*0.84*	0.2	*0.62*
**3**	0.3	*0.66*	-0.7	*0.41*	-0.6	*0.03*
**4**	-0.3	*0.73*	-2.3	*0.12*	-1.3	*<0.001*
**5**	-0.7	*0.43*	-2.9	*0.09*	-1.3	*<0.001*
**6**	-0.9	*0.22*	-2.1	*0.16*	-0.3	*0.25*
**7**	-0.9	*0.16*	-0.8	*0.73*	0.9	*0.01*
**8**	-0.8	*0.24*	0.0	*0.70*	1.5	*<0.001*
**9**	-0.7	*0.31*	0.3	*0.46*		
**10**	-0.6	*0.35*	0.3	*0.35*		
**11**	-0.5	*0.38*				
**12**	-0.4	*0.48*				

Table S2. Difference in Growth Rate Over Time According to Gestational Age Group, Between Infants With and Without Bronchopulmonary Dysplasia.

Bronchopulmonary dysplasia (BPD) is defined as requiring O_2 at 36 weeks postmenstrual age (PMA). Growth rate is reported in grams/kilogram/day. Data are shown as mean and 95% confidence interval. Analyses are adjusted for exact gestational age at birth, gender, center, necrotizing enterocolitis (NEC), and birth weight standard deviation score (BWSDS).

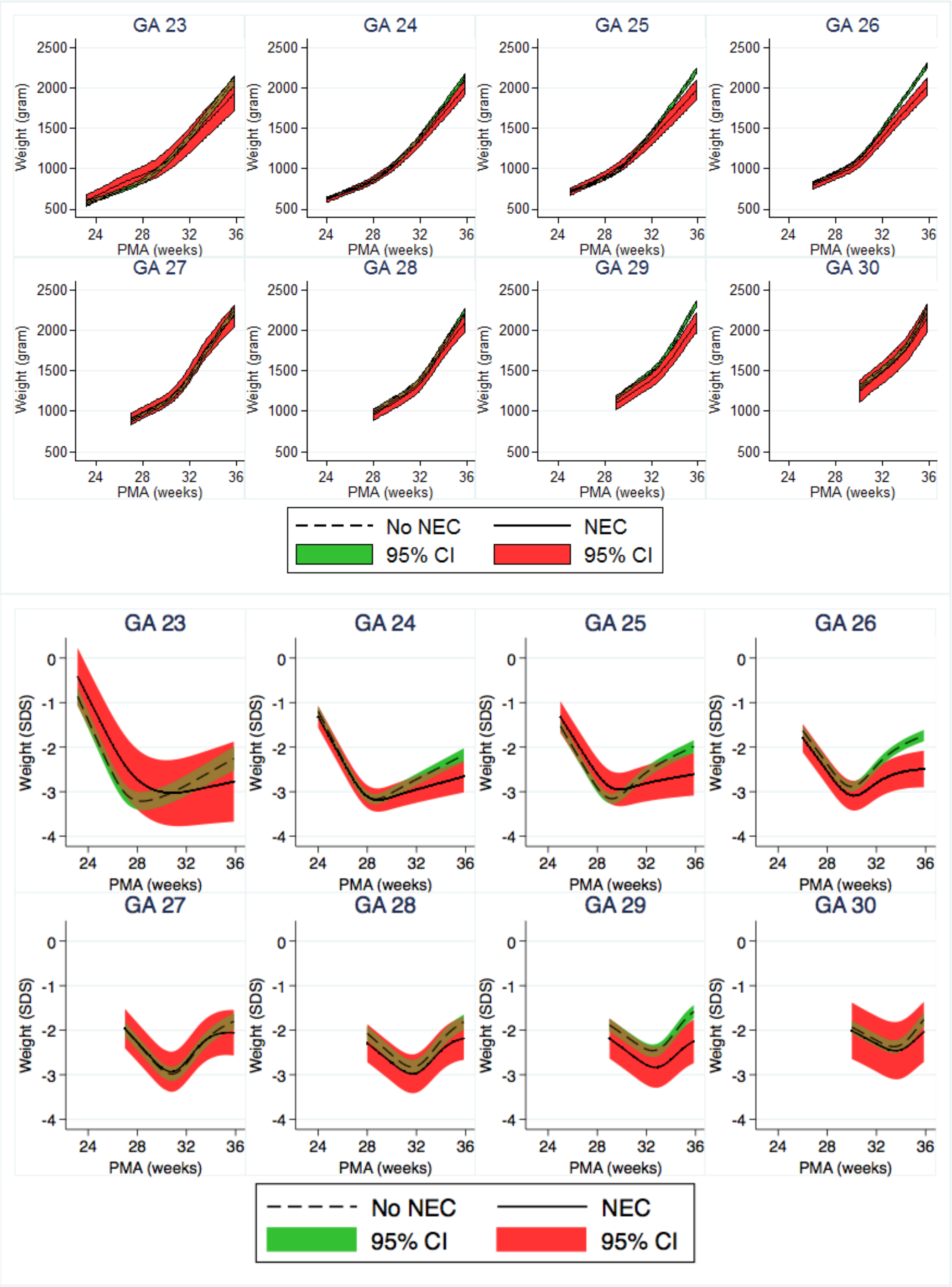

Figure S3. Postnatal Weight Development According to Gestational Age at Birth in Infants With and Without Necrotizing Enterocolitis (NEC).

Panel A shows postnatal weight development in grams. Panel B shows development in weight standard deviation score (SDS) according to Marsal[22]. Data are shown as mean and 95% confidence interval.

Gestational Age at birth (weeks)						
week of life	23-24		25-26		27-30	
	diff (g/kg/d)	p	diff (g/kg/d)	p	diff (g/kg/d)	p
**1**	1.7	*0.28*	0.5	*0.71*	0.2	*0.80*
**2**	1.6	*0.14*	-0.1	*0.92*	-0.2	*0.75*
**3**	1.6	*0.08*	-0.7	*0.39*	-0.5	*0.23*
**4**	1.4	*0.18*	-1.3	*0.19*	-0.8	*0.11*
**5**	0.8	*0.51*	-1.8	*0.09*	-1.1	*0.06*
**6**	-0.6	*0.51*	-2.2	*0.008*	-1.1	*0.01*
**7**	-2.1	*0.008*	-2.4	*<0.001*	-1.1	*0.02*
**8**	-2.9	*<0.001*	-2.5	*0.003*	-1.3	*0.03*
**9**	-3.1	*<0.001*	-2.3	*0.005*		
**10**	-2.9	*<0.001*	-2.1	*0.005*		
**11**	-2.5	*<0.001*				
**12**	-2.1	*0.001*				

Table S3. Difference in Growth Rate Over Time According to Gestational Age Group, Between Infants With and Without Necrotizing Enterocolitis (NEC).

Growth rate is shown in grams/kilograms/day. Data are shown as mean and 95% confidence interval.

Analyses are adjusted for exact gestational age at birth, gender and center.

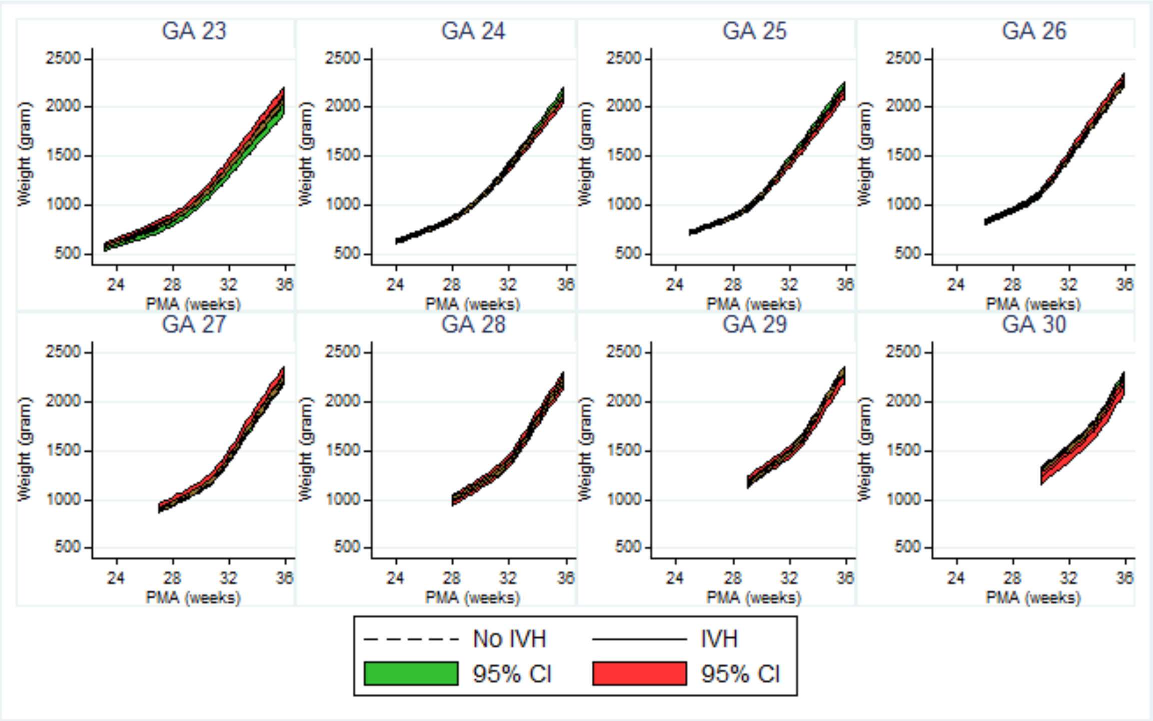

Figure S4. Postnatal Weight Development by Gestational Age at Birth in Infants With and Without Intraventricular Hemorrhage (IVH).

Postnatal weight development is shown in grams. Data are shown as mean and 95% confidence interval.

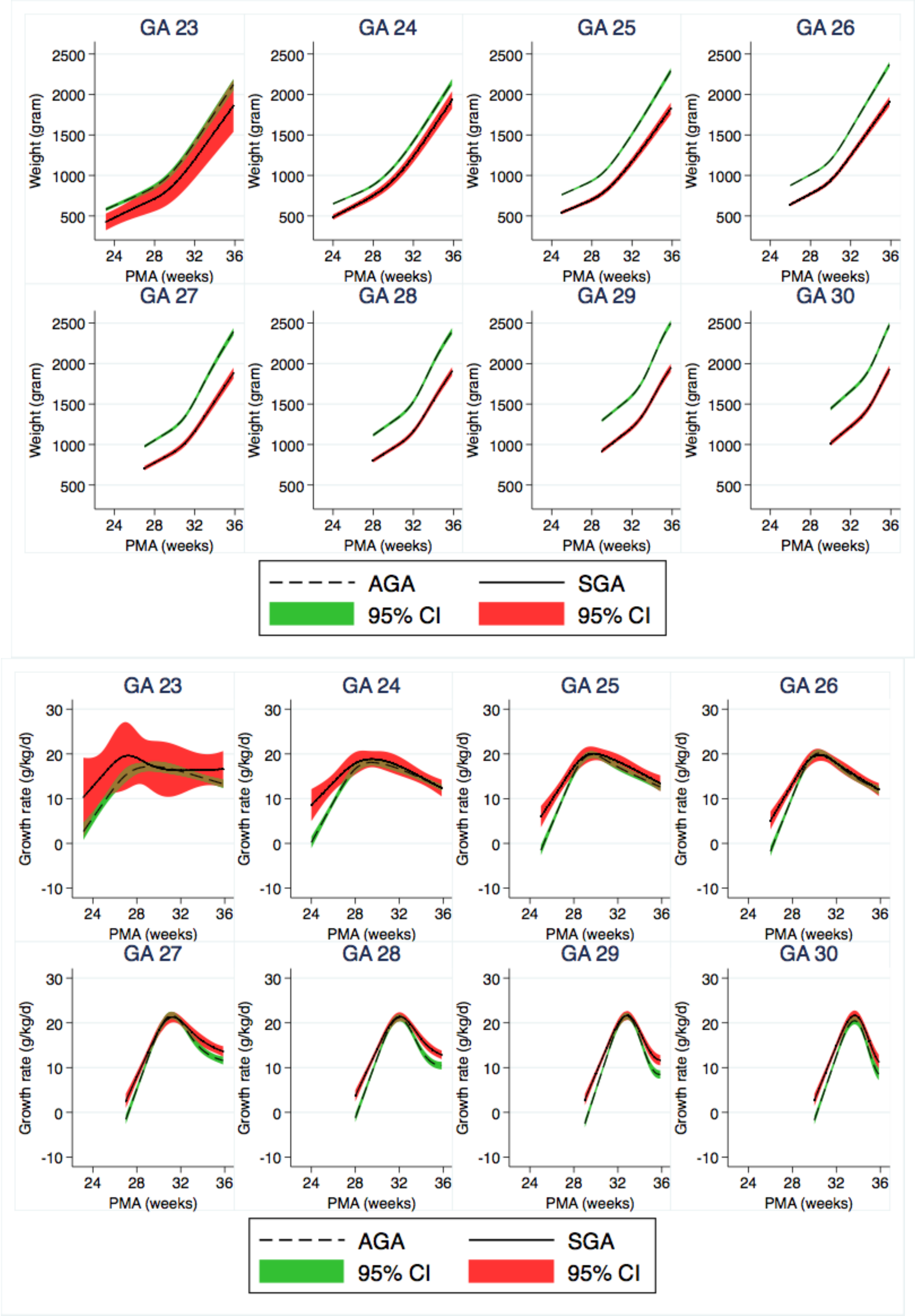

Figure S5. Postnatal Weight Development and Postnatal Growth Rate in Infants Born Small for Gestational Age (SGA) and Appropriate for Gestational Age (AGA) According to Gestational Age. Panel A shows postnatal weight development in grams. Panel B shows postnatal growth rate in grams/kilograms/day. Data are shown as mean and 95% confidence interval.

		Gestational Age at birth (weeks)					
		23-24		25-26		27-30	
		No BPD	BPD	No BPD	BPD	No BPD	BPD
No ROP	No NEC	2	23	80	145	618	149
	NEC	0	1	5	4	43	14
ROP	No NEC	65	282	166	411	205	138
	NEC	11	44	26	36	23	19

Table S4. Number of Infants Developing ROP, BPD, NEC and Combinations of these Morbidities by Gestational Age.
